# Patient-Oriented Questionnaires and Machine Learning for Rare Disease Diagnosis: A Systematic Review

**DOI:** 10.3390/jcm13175132

**Published:** 2024-08-29

**Authors:** Lea Eileen Brauner, Yao Yao, Lorenz Grigull, Frank Klawonn

**Affiliations:** 1 Department of Computer Science, Ostfalia University of Applied Sciences, 38302 Wolfenbuettel, Germany; le.brauner@ostfalia.de (L.E.B.); yao.yao1@ostfalia.de (Y.Y.); 2 Center for Rare Diseases Bonn (ZSEB), University Hospital of Bonn, 53127 Bonn, Germany; lorenz.grigull@ukbonn.de; 3 Helmholtz Centre for Infection Research, 38124 Braunschweig, Germany

**Keywords:** rare diseases, machine learning, diagnostic support

## Abstract

**Background:** A major challenge faced by patients with rare diseases (RDs) often stems from delays in diagnosis, typically due to nonspecific clinical symptoms or doctors’ limited experience in connecting symptoms to the underlying RD. Using patient-oriented questionnaires (POQs) as a data source for machine learning (ML) techniques can serve as a potential solution. These questionnaires enable patients to portray their day-to-day experiences living with their condition, irrespective of clinical symptoms. This systematic review—registered at PROSPERO with the Registration-ID: CRD42023490838—aims to present the current state of research in this domain by conducting a systematic literature search and identifying the potentials and limitations of this methodology. **Methods:** The review adheres to Preferred Reporting Items for Systematic Reviews and Meta-Analyses (PRISMA) guidelines and was primarily funded by the German Federal Ministry of Education and Research under grant no. 16DHBKI056 (ki4all). The methodology involved a systematic search across the databases PubMed, Semantic Scholar and Google Scholar, covering articles published until June 2023. The inclusion criteria encompass examining the use of POQs in diagnosing rare and common diseases. Additionally, studies that focused on applying ML techniques to the resulting datasets were considered for inclusion. The primary objective was to include English as well as German research that involved the generation of predictions regarding the underlying disease based on the information gathered from POQs. Furthermore, studies exploring identifying predictive indicators associated with the underlying disease were also included in the literature review. The following data were extracted from the selected studies: year of publication, number of questions in the POQs, answer scale in the questionnaires, the ML algorithms used, the input data for the ML algorithms, the performance of these algorithms and how the performance was measured. In addition, information on the development of the questionnaires was recorded. **Results:** This search retrieved 421 results in total. After one superficial and two comprehensive screening runs performed by two authors independently, we ended up with 26 studies for further consideration. Sixteen of these studies deal with diseases and ML algorithms to analyse data; the other ten studies provide contributing research in this field. We discuss several potentials and limitations of the evaluated approach. **Conclusions:** Overall, the results show that the full potential has not yet been exploited and that further research in this direction is worthwhile, because the study results show that ML algorithms can achieve promising results on POQ data; however, their use in everyday medical practice has not yet been investigated.

## 1. Introduction

According to the European Commission on Public Health [1], in the European Union (EU), up to 36 million people live with a RD. There is no standardised definition for RDs. Some definitions are based solely on the prevalence of a disease within a population, while others consider additional factors, such as the availability of suitable treatments or the disease’s severity. The most common definitions come from the EU, the United States of America, the United Kingdom and Japan. In the EU, a disease is considered rare if it does not affect more than 5 out of 10,000 people. More than 6000 RDs are present in the EU, varying in prevalence from a few patients to up to 245,000 patients. More detailed information on individual definitions of RDs can be found in [2,3,4,5].

RDs are medical challenges due to their rarity and lack of specificity of symptoms. Traditional diagnostic routines usually reach their limits here—a recent study [6] presented progress in this context. Using the publicly available Orphanet database [7] and its Orphanet epidemiological file, the authors estimated the cumulative prevalence at the point of RDs as a group. The population prevalence of RDs was estimated to be 3.5–5.9%, which equates to about 260–440 million individuals affected worldwide at any time. This reflects an enormous burden on the health system and affected families. A typical starting problem in therapy for RDs is usually a diagnostic delay, often caused by nonspecific clinical symptoms or lack of experience of the doctors consulted in linking symptoms with the underlying RD [8]. In general, most patients first consult a paediatrician (first indications for a disease often show up at an early stage of childhood) or a general practitioner, and are then referred to various specialists before an accurate diagnosis can be made. The results of the study [9] show that general practitioners have the weakest knowledge about RDs, while other specialists are better informed. Finding the right doctor commonly takes years and is often very frustrating for the patients [8].

Although people with RDs face many challenges, the diagnostic delay is still of utmost importance. Therefore, it is essential to develop alternative approaches to improve the accuracy and efficiency of diagnostic processes. One possible solution is to use intelligent systems that can provide diagnostic support. For example, in publication [10], the authors combined online search data for specific symptoms with national incidence data for RDs in China. They were able to recognise particular correlations. However, such query forecasts can also have their pitfalls. The Google Flu Trends [11] project tried something similar to predict flu epidemics, but this project was abandoned because the results did not behave as desired [12]. The authors in [10] clearly state that epidemiological data on RDs should still be included. So, we suggest collecting such epidemiological data on RDs using POQs. A review paper [13] on general diagnostic support systems for RDs is available, which declares that such systems can be based on data from questionnaires, images, laboratory values, or genetic data [14].

Moreover, ML integration has emerged as a powerful tool to improve diagnostic processes and decision making in medicine. ML, a subset of artificial intelligence, enables computer programs to autonomously identify intricate patterns and relationships within vast data sources, such as medical images, laboratory values, or genetic data [15]. It derives rules from these identified structures, facilitating subsequent predictions based on the learned patterns [16].

This paper focuses on integrating the patient’s perspective into the diagnostic process. Patients are experts in their own experiences and experiences with the disease. Their stories and experiences can provide valuable insights that can help to develop a more comprehensive understanding of rare diseases. One promising approach is to use patient-oriented questionnaires (POQs). These tailored tools are designed to capture patients’ day-to-day challenges and experiences, such as the struggle of opening a screw-top bottle, and can provide important indications for a diagnosis when shared by several patients with the same disease. POQs are user-friendly instruments that enable patients to portray their experiences in their daily lives, providing a holistic view beyond just clinical symptoms. Furthermore, incorporating ML in the automated analysis of POQs presents a promising frontier. ML algorithms can sift through qualitative data, identify patterns and extract meaningful insights. By harnessing patients’ expertise in recounting their experiences, ML can contribute to a deeper understanding of RDs, thereby streamlining the diagnostic process. To the best of the authors’ knowledge, a review paper encompassing the latest state of research in this specific domain is yet to be uncovered.

In this systematic review, we investigate the utility of POQs in combination with ML techniques in recognising RDs’ studies, that examine the use of POQs in diagnosing RDs and applying ML to the resulting datasets to generate predictions about the underlying disease. The aim of this review is to present the current state of research in this area and to identify the potentials and limitations that have been explored.

The relevance of this research is emphasised by the German National Action Plan for People with Rare Diseases (NAMSE) [17]. This action plan clearly states the need for improved diagnostic support for people with RDs. The integration of POQs and ML, as investigated in this systematic review, could make a significant contribution to the goal on improving diagnostic support for RDs.

## 2. Materials and Methods

### 2.1. Research Design

This paper discusses current challenges and solution approaches to recognising RDs using POQs and ML techniques. First, a systematic literature research is performed, following the guidelines of the PRISMA checklist [18], and the current state of research is presented. This is then used to identify the potentials and limitations of this methodology. Afterwards, the results are discussed with the help of a conclusion and an outlook.

### 2.2. Study Selection

The study selection process adhered to a systematic approach following the guidelines outlined in [18] and the PRISMA checklist. The systematic review was registered at PROSPERO [19] (Registration-ID: CRD42023490838). Afterwards, the following research question was developed based on the objectives.

**Research Question** To what extent has the systematic use of structured history-taking questions and questionnaires for diagnostic support been explored in medical research, and what evidence exists regarding the effectiveness and precision of these methods in supporting the diagnosis of RDs?

Based on the formulated research question, suitable search terms were then specified, which were used as search criteria for browsing online literature databases. For the research, English and German search terms were determined to cover the English and German literature. The combination of these then formed the following search queries, shown in Table 1.

These search terms were used to browse the PubMed [20], Semantic Scholar [21] and Google Scholar [22] databases. The literature search was performed by the author LEB. The time of the literature search, and thus also of the last retrieval of the current state of research, was June 2023. All other new developments are not taken into account in this work.

Table 2 gives the inclusion and exclusion criteria. Various publications, including original research articles and reviews, were considered for inclusion, provided they met the established criteria. For the administration of the literature, the open-source tool Zotero [23] was used. The first step involved the elimination of duplicates, which was supported by the Zotero tool. Then, three screening runs were performed, in which the literature was checked following the defined inclusion and exclusion criteria. The authors LEB and FK performed the screening of the literature; they worked independently. In the first run, only titles and abstracts were considered, while, in the subsequent runs, the full text of the selected publications was subject to an in-depth review.

### 2.3. Data Extraction

In order to generate a comprehensive overview, the following data were extracted from the selected studies: year of publication, number of questions in the questionnaires used, answer scale in the questionnaires, the ML algorithms used and the input data for the ML algorithms. In addition, information on the development of the questionnaires and performance indicators of the ML algorithms were recorded. The process of data extraction and preparation was performed by the author LEB.

### 2.4. Risk of Bias

The risk of bias in this paper can be summarised in the following aspects:

- Selection bias: a lack of comprehensive search strategy or inclusion criteria, leading to the exclusion of relevant studies. This can result in an incomplete synthesis of evidence and bias the conclusions drawn from the review.
- Publication and reporting bias: in the majority of publications, positive results are often emphasised, potentially overshadowing undisclosed negative results and rendering us unaware of their existence due to non-publication. This selective reporting may lead to an overestimation of treatment effects and intervention outcomes.
- Recall and knowledge bias: in certain studies, patients were requested to complete questionnaires post-diagnosis or post-treatment, potentially resulting in patients possessing enhanced knowledge about the disease. Additionally, participants may be tasked with recalling information from memory, which can lead to distorted responses if past events or experiences are inaccurately remembered.
- Methodological bias: when assessing ML algorithms, it is uncommon to evaluate quality based on a single measure. Certain studies solely mentioned accuracy (without specifying class distribution), while others exclusively presented recall (without precision or $F_{1}$-score). The upcoming Section 3.3.5 will delve into a more detailed explanation of performance measurement.

## 3. Results

### 3.1. Literature Search

**Figure 1:**
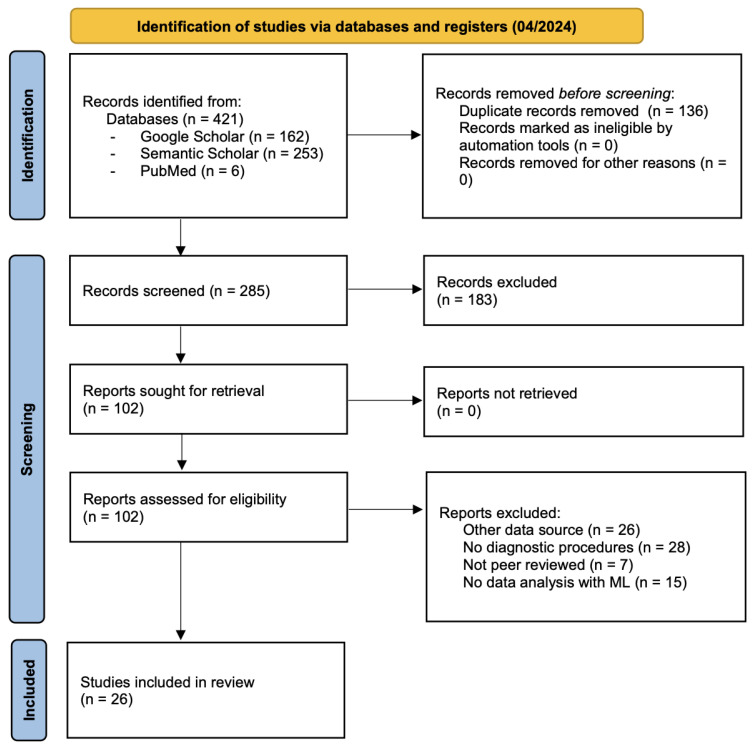
PRISMA 2020 flow diagram.

The search terms given in the previous section were used to browse the PubMed [20], Semantic Scholar [21] and Google Scholar [22] databases, which yielded 421 results in total. In Figure 1, the PRISMA flow diagram is shown, which illustrates the systematic literature research process. The German search terms yielded only one result. Beyond that, only English literature was retrieved. As we only deal with English literature, it is, of course, possible that the results might suffer from a language bias. However, we do not expect this to be significant, as the main language of science is English. Using the German language example, we can already see that only one German publication could be found. After removing duplicates, 285 publications remained.

In the next step, a first superficial check was performed using the titles and abstracts to filter which publications did not correspond to the research question. Afterwards, the remaining 102 publications were inspected more precisely, and the unsuitable ones were again excluded. This took two more screening runs. The reasons for the exclusion of the studies were diverse. Some of the studies screened used alternative data sources to the questionnaire data, for example, blood or urine values, genetic information, or medical images such as serial CT scans. Furthermore, some studies focused on something other than investigating diagnostic procedures, instead identifying the support needs of patients with a particular disease. Also, preprints that had yet to be peer reviewed were excluded from the analysis. In addition, publications that aimed to build a patient database based on questionnaire data but did not use ML techniques to analyse the data were not included.

After this comprehensive screening, 26 publications were identified for further consideration. Sixteen of these studies deal with diseases and use ML algorithms to analyse data from patient-oriented surveys [24,25,26,27,28,29,30,31,32,33,34,35,36,37,38,39]. The other ten studies cannot be applied one-to-one to detecting RDs, but they are nevertheless helpful in contributing to research in this field. They are also considered here because the field of RD detection with POQs has yet to be widely explored. A review of diagnostic systems for detecting RDs from 2020 [13] identified only two suitable publications in the literature that used POQs. This highlights the limited literature available in this field. Thus, studies were included that did not focus exclusively on patient experiences but also covered clinical symptoms. Ten of the twenty-six identified publications are studies that help answer the RQ from a different perspective ([1,8,9,40,41,42,43,44,45]). For example, they examine the acceptance of diagnostic support systems among physicians [41] or present the information needs for the diagnosis of selected RDs [9].

### 3.2. Literature Review

Table S1 in the supplement summarises the study characteristics. Across sixteen studies, six of these are studies that deal with RDs ([24,25,26,27,28,29]); seven studies deal with non-RDs like Parkinson, dementia, or infectious diseases ([30,31,32,33,34,35,36]). Furthermore, three publications were identified dealing with the recognition of psychosomatic disorders ([37,38,39]).

For questionnaire development, there were three predominant approaches [24,25,26,27,28,29,31,33,38]. The first conducted comprehensive patient interviews in advance, which were used to identify significant phenomena and extract the questions from the questionnaires. The second most used method was systematic literature research on the underlying disease [29,30,31,33,34,36,37]. Two other studies developed the questionnaires in collaboration with medical experts in the field of the disease ([32,33]). Ref. [38] did not develop questionnaires, but explored existing questionnaires. Refs. [35,39] did not specify the questionnaire development process.

For ML technologies, in addition to typical ML methods, we have also included nonlinear statistical methods like Logistic Regression (LR). LR was used in half of all studies ([24,25,26,27,28,30,33,37]) and was therefore the most common choice. It should be noted that the “Threshold” used in [36] is not an ML technique, but rather a fixed decision boundary. Refs. [38,39] do not specify the ML technologies used. The details can be found in Section 3.3.4 as well.

For data input for further data analysis, twelve studies focused only on POQs [24,25,26,27,28,32,33,34,37,39,39]. Ref. [30] additionally used a peak flow meter and lung sounds. Ref. [31] utilised an extra respiratory flow meter. Ref. [36] used POQs and diagnoses using spirometry. However, only [35] concentrated not mainly on POQs but passive digital markers based on electronic health record (EHR) data and patient-reported outcomes (the Quick Dementia Rating Scale).

Nine studies ([24,25,26,27,28,31,32,36,37]) developed a questionnaire and a POQ-based diagnostic tool to detect different diseases. Four studies [29,33,34,39] developed only a diagnostic questionnaire. Ref. [30] created a mobile tool with the help of questionnaires and electronic stethoscope to differentiate chronic obstructive pulmonary disease (COPD) and asthma (AS). Ref. [38] analysed existing questionnaires to find heterogeneity between mental disorders. Ref. [35] utilised the existing EHR data and patient-reported outcomes for the early detection of Alzheimer’s disease and related dementias.

### 3.3. Statistical Facts

#### 3.3.1. Chronological Trend

**Figure 2:**
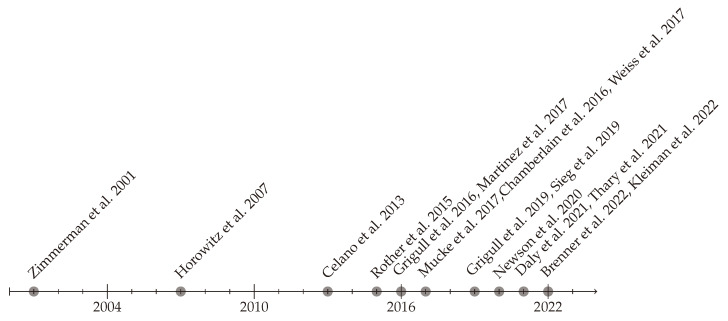
Timeline of the publications [24,25,26,27,28,29,30,31,32,33,34,35,36,37,38,39].

In Figure 2, the publications are listed on a timeline according to their year of publication. The figure shows that the number of publications on this topic has increased considerably in the last 10 years compared to the previous 10 years.

#### 3.3.2. Number of Questions

The number of questions to be answered in the questionnaires varies enormously. There are questionnaires containing under 10 ([31,36,37]), 10 to 20 ([29,33]), 20 to 40 ([26,28,34]), 40 to 50 ([24,27,28]), 50 to 100 ([25]) and over 100 questions ([39]). The number of questions present in [30,32,35,38] is not specified.

#### 3.3.3. Answer Scales

Different scales can be found, on the basis of which the questions can be answered. Mostly they were Likert scaled from 1 (“absolutely not true”) to 6 (“completely true”) or on a 3-point Likert scale (“yes”, “maybe”, “no”) ([24,25,27,28,31,33,36]). Some questionnaires only ask about the occurrence of phenomena in binary form ([29,31,34,36,37,39]). The answer scales in [26,30,32,35] are not specified.

#### 3.3.4. ML Techniques

The studies presented in the previous chapter use a number of ML techniques to extract diagnostic indications from the collected data. The distribution of these is displayed in Table 3.

#### 3.3.5. Performance

For the detection of a disease, the consideration of the evaluation metric *recall* plays a major role. A recall of 0.90 means that 90% of the people with the disease were actually identified as such by the diagnostic tool. It is thus important to maximise this indicator. Therefore, in the overview of the performance of the individual systems, the focus will be on the highest achieved recall. However, the relation to other metrics must always be considered. A system that simply identifies every subject as “affected” has a recall of 1, but does not provide valuable information. This can be remedied by the $F_{1}-$measure, which considers precision and recall together. Publications that do not achieve at least an $F_{1}-$measure of more than 0.5 or have not specified any other metrics are not discussed further, as an interpretation of the results is not possible.

The achieved recalls ranged from 0.69 to 0.98. Results of less than 0.80 were achieved by [36] and [33]. Refs. [25,28,34,39] reported good results between 0.81 and 0.90. Results well above 0.95 were achieved by [24,26,27,31].

## 4. Discussion

The process of discussing and interpreting the results was performed by the authors LEB, LG and FK.

### 4.1. Potentials

From the publications presented in the previous section, a range of potentials for the use of ML on questionnaire data could be identified. On the one hand, there are different possible applications for ML. *Predictive indicators* for certain diseases can be identified [40], for example by using logistic regression parameters. Likewise, classifiers can be used to *predict a possible diagnosis*. Furthermore, an advantage lies in the increase in efficiency. ML techniques are able to analyse large amounts of data and recognise patterns that are relevant for making a diagnosis but would be difficult for humans to identify [42]. The easy *accessibility* of questionnaires, for example by offering POQs via web applications, allows participation without demographic restrictions. Thus, even patients with potentially limited access to specialised medical institutions can be reached. Furthermore, questionnaires are *easy to use.* They can be completed directly by patients or their parents/caregivers, making them much easier to use in practice [42]. Research on RDs can also benefit from the application of ML. *New knowledge* about RDs can be gained by identifying predictive indicators. This updates the state of research with regard to previously unknown correlations and risk factors. Another advantage is the objectivity of ML methods, as they can bypass subjective human judgements and make *objective decisions*. However, this is only valid if the training data is actually unbiased and representative. Studies like Ref. [41] show that there is already a high level of acceptance among medical professionals for such technologies and procedures to support diagnosis. In addition, costs can be saved because the potential for the early detection of RDs can reduce long and expensive diagnostic procedures. Especially expensive invasive examinations can then be used in a more targeted manner after the system has suggested a diagnosis. Further studies, such as [44], that investigate the massive delay in RD diagnosis, point out that the most important aspects are the improvement in the medical diagnosis system through better coordination between different health care institutions and the use of digital tools such as questionnaires in combination with ML techniques.

Furthermore, the questionnaire data obtained offer an added value not only for individual patients, whose diagnostic process can be accelerated, but for the population as a whole. The analysis of aggregated questionnaire data can help uncover long-term health trends and provide insights into the general population health. This can assist in the development of public health strategies and interventions.

### 4.2. Scope and Limitations

The use of POQs in combination with ML for the diagnosis of RDs holds promising potential, but also comes with a range of challenges. One of the key challenges is the *availability of high-quality and sufficient data*. POQs capture patients’ subjective experiences and symptoms, which can be diverse and complex. To achieve meaningful results, it is necessary to include a sufficiently large and representative sample of patients who actually have an experience with the disease. However, the *limited number* of patients with RDs can complicate data collection and hinder the development of robust diagnostic models [14].

The questionnaires from the studies discussed here often consist of very *many questions* in order to provide a comprehensive snapshot of the patients suffering. People who suffer from pain disorders or mental illnesses have to invest a lot of energy and concentration to complete these questionnaires faithfully. To address this disadvantage, the study authors [43] developed an approach to minimise the number of questions to be answered, still trying to gather enough information. An algorithm was developed that analyses a patients previous response pattern. Then, questions that maximise the information gain are specifically and dynamically selected to provide strong evidence for or against a diagnostic tendency.

The use of patient data for research purposes, especially in the field of health, requires careful consideration of ethical and *data protection aspects*. Patients must be informed about how their data will be used, the risks and benefits involved, and how their privacy will be protected. A key concern relates to the anonymisation and pseudonymisation of data to protect the identity of patients. In particular in the case of RDs, it can be easier to identify individual patients from limited amounts of data, increasing the risk of abuse.

Another obstacle is the *heterogeneity of symptoms* and the courses of RDs. Often, patients with the same RD present with different symptoms, which makes it difficult to develop a uniform diagnostic methodology. The diversity of symptoms therefore requires an individualised approach and consideration of a wide range of factors even within a single disease.

### 4.3. Research Question Review

In response to the research question regarding the systematic utilisation of structured history-taking questions and questionnaires for diagnostic support, this review reveals significant exploration in medical research. There are indications that, with appropriately trained and validated ML models, this approach can definitely serve as a powerful diagnostic support tool for various diseases. However, the extent to which this approach can be fully integrated and its impact on accelerating the diagnosis of RDs requires further investigation and specific studies. In particular, the actual use of the proposed systems in the daily clinical routine needs to be examined.

## 5. Conclusions

In summary, the study of the integration of POQs and ML techniques in the field of medical diagnosis reveals both great progress and ongoing challenges. The comprehensive analysis of several studies showed the potential of using patient data in combination with ML algorithms to support the diagnosis of a wide range of diseases, including both rare and common physical and mental disorders. These studies have shown that ML models, when appropriately trained and validated, can provide a helpful first impulse towards diagnosis and support medical professionals in their decision-making processes. Despite the promising results, there are still some challenges on the way to effectively implementing this innovative approach. One notable challenge is the availability and quality of data. Especially for RDs, obtaining comprehensive and representative datasets remains a challenge due to the limited number of cases. There is also the question of accountability when these systems make errors in diagnosis. It is important to note that such systems can only serve as a support and not as a substitute for the process of finding a diagnosis, for which a high level of acceptance among medical staff has already been recorded.

The future of RD diagnosis using patient-centred questionnaires and ML is promising, with steady advances in both the medical and technological fields. Already, some of the studies presented here used data from patient questionnaires combined with other data. One promising development concerns the integration of patient data from different sources, such as image data, laboratory results and genetic analyses, and questionnaire data, to create more comprehensive and accurate diagnostic models. A second important research potential is the examination of the use of these systems in the daily clinical routine. From our perspective, the results show that the full potential has not yet been exploited and that further research in this direction is worthwhile.

## Figures and Tables

**Table 1:** Search queries.

**questionnaire**
*AND*
(**patient oriented questionnaire** *OR* **patient reported outcome** *OR* **patient experience** *OR* **patient perspective**)
*AND*
(**machine learning** *OR* **artificial intelligence** *OR* **data mining**)
*AND*
(**diagnostic support** *OR* **clinical decision making**)
*AND*
(**rare disease** *OR* **orphan disease**)

**Table 2:** Inclusion and exclusion criteria.

Inclusion Criteria	Exclusion Criteria
Examination of the use of POQs in diagnosing (rare) diseases Application of ML techniques to the resulting datasets Generation of predictions about the underlying disease Identification of predictive indicators about the underlying disease	Utilisation of other data sources than POQs Other objectives than diagnostic procedures Not peer reviewed articles No use of ML techniques for data analysis

**Table 3:** ML techniques.

Model	Abs. Feq	Rel. Freq	Publications
Naive Bayes	4	25%	[24,26,27,32]
LDA	6	38%	[24,25,26,27,28,32]
QDA	1	6%	[32]
Decision Tree	1	6%	[33,34]
Random Forest	5	31%	[24,25,26,28,31]
Logistic Regression	8	50%	[24,25,26,27,28,30,33,37]
SVM	6	38%	[24,25,26,27,28,34]
KNN	4	25%	[24,26,27,32]
Fuzzy RB	3	6%	[27]
MLP	1	19%	[27,33,34]
Fusion	5	31%	[24,25,26,27,28]
Threshold	1	6%	[36]

## Supplementary Materials

The following supporting information can be downloaded at: https://www.mdpi.com/article/10.3390/jcm13175132/s1, Table S1: summary of literature reviews.

## References

[1] European Commission on Public Health Rare Diseases. https://health.ec.europa.eu/non-communicable-diseases/expert-group-public-health/rare-diseases_en.

[2] United States Congress Rare Disease Act of 2002. https://www.govinfo.gov/content/pkg/PLAW-107publ280/html/PLAW-107publ280.htm.

[3] Rare diseases centre-Venetian Region-Italy. Rare Diseases: What Are We Talking About?. http://malattierare.regione.veneto.it/inglese/dicosaparliamo_ing.php.

[4] European Commission Useful Information on Rare Diseases from an EU Perspective. https://ec.europa.eu/health/ph_information/documents/ev20040705_rd05_en.pdf.

[5] Baldovino S., Moliner A.M., Taruscio D., Daina E., Roccatello D. (2016). Rare Diseases in Europe: From a Wide to a Local Perspective. Isr. Med. Assoc. J. IMAJ.

[6] Nguengang Wakap S., Lambert D.M., Olry A., Rodwell C., Gueydan C., Lanneau V., Murphy D., Le Cam Y., Rath A. (2020). Estimating cumulative point prevalence of rare diseases: Analysis of the Orphanet database. Eur. J. Hum. Genet. EJHG.

[7] Institut National de la Santé et de la Recherche Médicale Orphanet. https://www.orpha.net/.

[8] Grigull L. (2021). Seltene Erkrankungen und der lange Weg zur Diagnose.

[9] Vandeborne L., van Overbeeke E., Dooms M., de Beleyr B., Huys I. (2019). Information needs of physicians regarding the diagnosis of rare diseases: A questionnaire-based study in Belgium. Orphanet J. Rare Dis..

[10] Zhang L., Jin Y., Li J., He Z., Zhang D., Zhang M., Zhang S. (2023). Epidemiological research on rare diseases using large-scale online search queries and reported case data. Orphanet J. Rare Dis..

[11] Google Inc. Google Flu Trends. https://en.wikipedia.org/wiki/Google_Flu_Trends.

[12] Lazer D., Kennedy R., King G., Vespignani A. (2014). Big data. The parable of Google Flu: Traps in big data analysis. Science.

[13] Schaaf J., Sedlmayr M., Schaefer J., Storf H. (2020). Diagnosis of Rare Diseases: A scoping review of clinical decision support systems. Orphanet J. Rare Dis..

[14] Topol E.J. (2019). High-performance medicine: The convergence of human and artificial intelligence. Nat. Med..

[15] Vasey B., Ursprung S., Beddoe B., Taylor E.H., Marlow N., Bilbro N., Watkinson P., McCulloch P. (2021). Association of Clinician Diagnostic Performance with Machine Learning-Based Decision Support Systems: A Systematic Review. JAMA Netw. Open.

[16] Liu Y., Chen P.H.C., Krause J., Peng L. (2019). How to Read Articles That Use Machine Learning: Users’ Guides to the Medical Literature. JAMA.

[17] National Action League for People with Rare Diseases (2013). National Plan of Action for People with Rare Diseases. https://www.namse.de/fileadmin/user_upload/downloads/National_Plan_of_Action.pdf.

[18] Page M.J., McKenzie J.E., Bossuyt P.M., Boutron I., Hoffmann T.C., Mulrow C.D., Shamseer L., Tetzlaff J.M., Akl E.A., Brennan S.E. (2021). The PRISMA 2020 statement: An updated guideline for reporting systematic reviews. BMJ.

[19] University of York PROSPERO. https://www.crd.york.ac.uk/PROSPERO/.

[20] National Library of Medicine PubMed. https://pubmed.ncbi.nlm.nih.gov/.

[21] Allen Institute for AI. Semantic Scholar. https://www.semanticscholar.org/.

[22] Google Inc. Google Scholar. https://www.scholar.google.com/.

[23] Corporation for Digital Scholarship Zotero. https://www.zotero.org/.

[24] Grigull L., Lechner W., Petri S., Kollewe K., Dengler R., Mehmecke S., Schumacher U., Lücke T., Schneider-Gold C., Köhler C. (2016). Diagnostic support for selected neuromuscular diseases using answer-pattern recognition and data mining techniques: A proof of concept multicenter prospective trial. Bmc Med. Inform. Decis. Mak..

[25] Grigull L., Mehmecke S., Rother A.K., Blöß S., Klemann C., Schumacher U., Mücke U., Kortum X., Lechner W., Klawonn F. (2019). Common pre-diagnostic features in individuals with different rare diseases represent a key for diagnostic support with computerized pattern recognition?. PLoS ONE.

[26] Mücke U., Klemann C., Baumann U., Meyer-Bahlburg A., Kortum X., Klawonn F., Lechner W.M., Grigull L. (2017). Patient’s Experience in Pediatric Primary Immunodeficiency Disorders: Computerized Classification of Questionnaires. Front. Immunol..

[27] Rother A.K., Schwerk N., Brinkmann F., Klawonn F., Lechner W., Grigull L. (2015). Diagnostic Support for Selected Paediatric Pulmonary Diseases Using Answer-Pattern Recognition in Questionnaires Based on Combined Data Mining Applications—A Monocentric Observational Pilot Study. PLOS ONE.

[28] Sieg A.L., Martin Das A., Maria Muschol N., Köhn A., Lampe C., Kortum X., Mehmecke S., Blöß S., Lechner W., Klawonn F. (2019). Künstliche Intelligenz zur diagnostischen Unterstützung ausgewählter seltener lysosomaler Speichererkrankungen: Ergebnisse einer Pilotstudie. Klin. Padiatr..

[29] Daly R.P., Jalbert J.J., Keith S., Symonds T., Shammo J. (2021). A novel patient-reported outcome instrument assessing the symptoms of paroxysmal nocturnal hemoglobinuria, the PNH-SQ. J. Patient-Rep. Outcomes.

[30] Chamberlain D.B., Kodgule R., Fletcher R.R. A mobile platform for automated screening of asthma and chronic obstructive pulmonary disease. Proceedings of the Annual International Conference of the IEEE Engineering in Medicine and Biology Society, IEEE Engineering in Medicine and Biology Society. Annual International Conference.

[31] Martinez F.J., Mannino D., Leidy N.K., Malley K.G., Bacci E.D., Barr R.G., Bowler R.P., Han M.K., Houfek J.F., Make B. (2017). A New Approach for Identifying Patients with Undiagnosed Chronic Obstructive Pulmonary Disease. Am. J. Respir. Crit. Care Med..

[32] Thary H.H., Zidan K.A. (2021). A Framework Questionnaire for Diagnosing Infectious Disease Using Machine Learning Techniques. Iop Conf. Ser. Mater. Sci. Eng..

[33] Horowitz N., Moshkowitz M., Halpern Z., Leshno M. (2007). Applying data mining techniques in the development of a diagnostics questionnaire for GERD. Dig. Dis. Sci..

[34] Brenner A., Plagwitz L., Fujarski M., Warnecke T., Varghese J. (2022). Utilizing a Non-Motor Symptoms Questionnaire and Machine Learning to Differentiate Movement Disorders. Stud. Health Technol. Inform..

[35] Kleiman M.J., Plewes A.D., Owora A., Grout R.W., Dexter P.R., Fowler N.R., Galvin J.E., Miled Z.B., Boustani M. (2022). Digital detection of dementia (D3): A study protocol for a pragmatic cluster-randomized trial examining the application of patient-reported outcomes and passive clinical decision support systems. Trials.

[36] Weiss G., Steinacher I., Lamprecht B., Kaiser B., Mikes R., Sator L., Hartl S., Wagner H., Studnicka M. (2017). Development and validation of the Salzburg COPD-screening questionnaire (SCSQ): A questionnaire development and validation study. NPJ Prim. Care Respir. Med..

[37] Celano C.M., Suarez L., Mastromauro C., Januzzi J.L., Huffman J.C. (2013). Feasibility and utility of screening for depression and anxiety disorders in patients with cardiovascular disease. Circ. Cardiovasc. Qual. Outcomes.

[38] Newson J.J., Hunter D., Thiagarajan T.C. (2020). The Heterogeneity of Mental Health Assessment. Front. Psychiatry.

[39] Zimmerman M., Mattia J.I. (2001). A self-report scale to help make psychiatric diagnoses: The Psychiatric Diagnostic Screening Questionnaire. Arch. Gen. Psychiatry.

[40] Blöß S., Klemann C., Rother A.K., Mehmecke S., Schumacher U., Mücke U., Mücke M., Stieber C., Klawonn F., Kortum X. (2017). Diagnostic needs for rare diseases and shared prediagnostic phenomena: Results of a German-wide expert Delphi survey. PLoS ONE.

[41] Patrzyk S., Bielecki W., Woźniacka A. (2022). A study of attitudes among Polish dermatologists and dermatology trainees regarding modern technologies in medicine. Postep. Dermatol. Alergol..

[42] Forsting M. (2017). Machine Learning Will Change Medicine. J. Nucl. Med. Off. Publ. Soc. Nucl. Med..

[43] Kortum X., Grigull L., Lechner W., Klawonn F., Adams N., Tucker A., Weston D. (2017). A Dynamic Adaptive Questionnaire for Improved Disease Diagnostics. Advances in Intelligent Data Analysis XVI.

[44] Isono M., Kokado M., Kato K. (2022). Why does it take so long for rare disease patients to get an accurate diagnosis?—A qualitative investigation of patient experiences of hereditary angioedema. PLoS ONE.

[45] Buist A.S., McBurnie M.A., Vollmer W.M., Gillespie S., Burney P., Mannino D.M., Menezes A.M.B., Sullivan S.D., Lee T.A., Weiss K.B. (2007). International variation in the prevalence of COPD (the BOLD Study): A population-based prevalence study. Lancet.

